# LRP1 Shedding in Ricin-Induced Lung Injury: A Cell-Specific Response to Toxin Exposure

**DOI:** 10.3390/ijms26125448

**Published:** 2025-06-06

**Authors:** Anita Sapoznikov, Yentl Evgy, Moshe Aftalion, Reut Falach

**Affiliations:** Department of Biochemistry and Molecular Genetics, Israel Institute for Biological Research, Ness-Ziona 74100, Israel; anitas@iibr.gov.il (A.S.); yentle@iibr.gov.il (Y.E.); moshea@iibr.gov.il (M.A.)

**Keywords:** LRP1, ricin, intranasal, lungs, fibroblasts

## Abstract

Ricin is a highly potent toxin that causes severe lung injury upon inhalation by initiating a complex cascade of cellular responses that ultimately leads to cell death. The low-density lipoprotein receptor-related protein 1 (LRP1) is a multifunctional receptor involved in various physiological processes, including ricin-mediated toxicity. This study explores the role of LRP1 shedding in the development of ricin-induced lung injury. Analysis of bronchoalveolar lavage fluid (BALF) from ricin-intoxicated mice and swine showed a significant increase in soluble LRP1 (sLRP1) levels, whereas serum LRP1 levels remained largely unchanged, suggesting the lungs are the primary source of sLRP1 release. In vitro assays demonstrated the formation of ricin-sLRP1 complexes, indicating that sLRP1 in BALF retained ricin-binding capability. Flow cytometric analysis of lung cells revealed a reduction in both the percentage and total number of LRP1-expressing cells following ricin exposure. Further investigation of specific lung cell populations showed that alveolar epithelial type II (AT-II) cells, despite experiencing significant injury, exhibited minimal LRP1 shedding. No shedding of LRP1 occurred in neutrophils. In contrast, fibroblasts, which were resistant to ricin-induced cell death, exhibited increased shedding of LRP1 and a corresponding decrease in membrane-bound LRP1 expression. This shedding of the LRP1 ectodomain was mediated by metalloproteinases. Immunohistochemical staining further confirmed decreased LRP1 expression in fibroblasts from ricin-exposed mice. Macrophages also showed substantial LRP1 shedding, despite undergoing significant depletion. These findings highlight the complex cell-specific nature of LRP1 shedding in response to ricin intoxication and suggests the potential role of LRP1 in modulation of cellular susceptibility and resistance to ricin-induced lung injury.

## 1. Introduction

The castor oil plant, Ricinus communis, harbors seeds containing the toxin ricin, a potent type II ribosome-inactivating protein (RIP). This toxin’s potential as a biological threat agent stems from its ubiquitous availability and relatively uncomplicated extraction process [1,2]. Upon inhalation, ricin elicits a severe pulmonary response characterized by a marked neutrophilic infiltration and a concomitant cytokine storm. These inflammatory events are accompanied by the development of significant edema in both the alveolar spaces and pulmonary interstitium. The cumulative effect of these pathological changes is profound respiratory dysfunction, which, if unmitigated, can progress to fatal respiratory failure [3,4,5]. In our prior investigation, we have demonstrated that the clinical presentation of respiratory injury caused by ricin intoxication in porcine model aligns completely with the established diagnostic parameters for acute respiratory distress syndrome (ARDS) [6].

Ricin is composed of two distinct polypeptide chains: A (RTA) and B (RTB). This heterodimeric structure is crucial to its function and toxicity. The A chain, or RTA, serves as the catalytic subunit. Its primary action is to depurinate a specific adenosine within the GAGA sequence of the sarcin-ricin loop in the 28S rRNA molecule [7,8]. This precise modification effectively halts protein synthesis, ultimately leading to cell death [9]. The B chain, or RTB, facilitates the toxin’s entry into cells. As a lectin, RTB belongs to a family of carbohydrate-binding proteins. It exhibits a high affinity for specific sugar structures, particularly terminal galactose and N-acetyl-galactosamine residues. This binding specificity allows ricin to attach to a wide array of cell surface molecules containing these sugar moieties, including various glycoproteins and glycolipids [10,11,12].

Ricin is a member of the AB toxin family, a group of potent biological agents produced by various bacterial pathogens and plants. Numerous toxins within the AB family have been found to interact with specific membrane receptors, a characteristic that facilitates their entry into target cells. This receptor specificity is exemplified by several well-studied toxins, the Cholera toxin that exhibits high-affinity binding to the GM1 ganglioside [13], Shiga toxin, which specifically binds to the globotriaosylceramide (Gb3) receptor [14,15] and Anthrax toxin that interacts with two known receptors, tumor endothelial marker 8 (TEM8) and capillary morphogenesis protein 2 (CMG2) [16].

There has been a wide consensus as to the non-selective nature of ricin binding to all cell-surface glycoproteins or glycolipids containing a terminal galactose link [17,18,19,20]. This would imply that numerous cell surface structures could serve as potential ricin receptors, facilitating its cellular uptake. However, recent research has challenged this notion and revealed intriguing patterns of ricin binding and activity following pulmonary exposure in mice. It has been found that ricin exhibits differential binding patterns across various lung cell populations and the level of ricin-induced depurination varies significantly among these cell types [5,21]. These findings suggest a more selective mechanism of ricin entry into cells than previously believed and suggest the presence of specific cell-surface receptors for ricin. Accordingly, LRP1 has been identified as a principal target molecule for ricin binding [22]. In this context, LRP1 has also been identified as a specific receptor for Pseudomonas aeruginosa exotoxin A (PEA) [23].

LRP1 is a versatile endocytic receptor responsible for the internalization of an extensive and diverse array of 40 extracellular ligands. The remarkable capacity of LRP1 to recognize and internalize such a wide spectrum of molecules underscores its critical role in various cellular processes and physiological functions including lipoprotein metabolism, cell signaling, and inflammation [24,25,26,27]. LRP1 is a member of the low-density lipoprotein receptor family and is widely expressed in various cell types, including macrophages, smooth muscle cells and epithelial cells [28,29,30]. LRP1 is synthesized as a 600-kilodalton (kDa) precursor transmembrane glycoprotein. This precursor undergoes proteolytic cleavage in the trans-Golgi network, resulting in the formation of a mature receptor composed of two non-covalently associated subunits: a large extracellular α-chain (approximately 515 kDa) and a smaller transmembrane β-chain (approximately 85 kDa) [29,31,32]. The α-chain of LRP1 is responsible for ligand binding and is characterized by its modular structure, which includes: LDLa, EGF-like and complement-type repeats. The β-chain structure includes an extracellular portion that is non-covalently associated with the α-chain, a single transmembrane domain and an intracellular tail [24].

Cumulative evidence indicates that the ectodomain of LRP-1 undergoes proteolytic cleavage at the cell surface, in several pathological conditions, including atherosclerosis, neuroinflammation, Alzheimer’s disease, lung inflammation and acute respiratory distress syndrome (ARDS) [33,34,35,36,37,38]. This mechanism results in the release of a soluble form of the receptor, termed sLRP-1, into the extracellular space. The shedding of LRP1 is mediated primarily by metalloproteinases that cleave the receptor at specific sites near the cell membrane, liberating the large extracellular domain (the α-chain and the extracellular domain of the β-chain) while leaving the transmembrane and intracellular portions intact. sLRP-1 retains many of the ligand-binding properties of the full-length receptor [39,40]. The presence of elevated sLRP-1 levels in biological fluids during inflammatory states suggests its potential utility as a biomarker for acute lung inflammation. Moreover, the released sLRP-1 may play functional roles in the inflammatory process, possibly by acting as a decoy receptor or by directly interacting with other inflammatory mediators in the extracellular space [37,38].

In this paper, we investigated the shedding of LRP1 in the context of ricin-induced lung inflammation and explored its physiological significance. Given the role of LRP1 in ricin internalization, we hypothesized that the shedding of LRP1 could serve two potential functions. Primarily, it might act as a protective mechanism, reducing cellular uptake of ricin by decreasing the number of available receptors on the cell surface of susceptible cells. This could potentially limit the ability of the toxin to enter cells and cause damage, thereby mitigating the severity of ricin poisoning. Alternatively, we considered the possibility that the released sLRP1 might originate from lung cells that are not directly relevant or resistant to ricin intoxication. In this scenario, the shedding of LRP1 might primarily play a role in modulating the inflammatory response itself, rather than directly affecting ricin internalization. This hypothesis is supported by studies in other inflammatory contexts that have demonstrated the immunomodulatory properties of sLRP1 [33,34,38].

By investigating these possibilities, we aim to elucidate the complex interplay between LRP1 shedding, ricin toxicity, and the inflammatory response in the lung. Understanding the source and function of sLRP1 in this context could provide valuable insights into the pathophysiology of ricin-induced lung injury and potentially facilitate the development of novel therapeutic strategies for ricin poisoning and other forms of acute lung inflammation.

## 2. Results

### 2.1. LRP1 Shedding in the Lungs Following Pulmonary Exposure to Ricin

**Figure 1:**
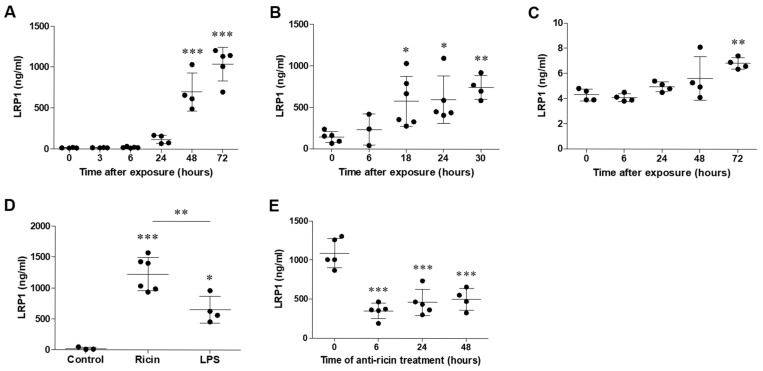
Soluble LRP1 (sLRP1) levels in BALF of mice and swine following ricin pulmonary intoxication. Mice and swine were exposed to ricin via intranasal (2LD_50_, 10 µg/kg) and intratracheal (2LD_50_, 3 µg/kg) route, respectively. BALF was collected at different time points post exposure. The levels of sLRP1 in (**A**) mouse, (**B**) swine BALF and (**C**) mouse serum at different time points post-ricin exposure. (**D**) Comparative analysis of sLRP1 levels in mouse BALF 72 h post-intranasal exposure to ricin or LPS (4 µg/kg). (**E**) The effect of anti-ricin antibody treatment, intravenously administered (100 µL, 60 mg/mL) at different time points post-ricin exposure, on sLRP1 levels in mouse BALF collected at 72 h post exposure. Data are presented as means ± SD (*n* = 3–6), each point represents an individual mouse. Statistical significance was determined using one-way ANOVA followed by Tukey’s multiple comparison post-hoc test. * *p* < 0.05, ** *p* < 0.01, *** *p* < 0.001 compared to control mice.

To determine whether LRP1 shedding occurs in the lungs following pulmonary exposure to ricin, we analyzed BALF samples for the presence of LRP1 in ricin-intoxicated mice. Mice and swine were exposed to a lethal dose of ricin (2LD_50_) intranasally or intratracheally, respectively, and BALF samples were collected at various time points post-exposure. In mice, we observed a gradual increase in LRP1 levels in the BALF following ricin exposure. This increase became apparent at 24 h post-exposure and continued to rise over time at 48- and 72-h post-exposure (Figure 1A), suggesting a potential correlation between the progression of ricin-induced lung injury and the extent of LRP1 shedding. Consistent with this observation in the mouse model, BALF samples collected from intratracheally exposed swine also exhibited significantly higher levels of LRP1 following ricin exposure compared to samples collected prior to intoxication. An initial significant increase in LRP1 levels was detected as early as 18 h post-exposure and its level kept increasing until 30 h post-exposure (Figure 1B).

In oppose to the BALF, analysis of LRP1 level in the serum in ricin-intoxicated mice showed largely unchanged concentration of this protein for the majority of the observation period. A significant increase in LRP1 in the serum was detected only at 72 h post-exposure (Figure 1C). The marked difference between the BALF and serum LRP1 levels, particularly the 170-fold higher concentration in BALF at 72 h post-exposure, strongly suggested that the primary source of the soluble LRP1 were the lungs. To evaluate the extent of LRP1 shedding in the lungs following pulmonary ricin exposure, we compared its intensity to that observed in a well-established model of acute pulmonary inflammation. To this end, we employed a lipopolysaccharide (LPS)-induced acute lung injury model, in which mice were intranasally administered with a lethal dose (4 mg/kg) of LPS. This exposure resulted in the development of severe widespread pneumonia, leading to mortality between 4 and 7 days following LPS administration. We compared the levels of LRP1 in the lungs of mice exposed to ricin or LPS at 72 h post-exposure, a time point that represents a critical stage in the progression of both types of lung injury. Notably, LRP1 level in ricin-exposed lungs was approximately 2-fold higher than this observed in LPS-exposed lungs (1127 ± 110.4 versus 653 ± 109.2 ng/mL, respectively, Figure 1D). This substantial difference suggested that the process of LRP1 shedding was more intense in ricin-induced lung injury than in LPS-induced lung inflammation, even though both models resulted in severe pulmonary damage and mortality at approximately the same time-points.

To further clarify the relationship between ricin-induced lung injury and LRP1 shedding, we examined the effect of anti-ricin antibody treatment, using polyclonal neutralizing antibodies [41], on soluble LRP1 levels in the BALF of ricin-exposed mice. Mice were exposed to a lethal dose of ricin via pulmonary route and subsequently treated with an anti-ricin antibody preparation at 6, 24- or 48-h post-exposure. BALF samples were collected from all groups at 72 h post-exposure and assessed for LRP1 levels. Administration of anti-ricin antibodies resulted in a significant decrease in BALF LRP1 levels across all treatment time points (Figure 1E). The consistent reduction in LRP1 across different treatment time points might suggest that the antibody intervention was effective in mitigating LRP1 shedding even when administered at later stages of ricin intoxication. Despite the significant decrease in soluble LRP1 levels following antibody treatment, it was important to note that these levels remained significantly higher compared to those observed in control mice (Figure 1A). This sustained increase in LRP1 levels, albeit diminished, indicated that while antibody treatment substantially reduced LRP1 shedding, it did not completely restore the lung environment to pre-exposure conditions within the 72-h timeframe.

**Figure 2:**
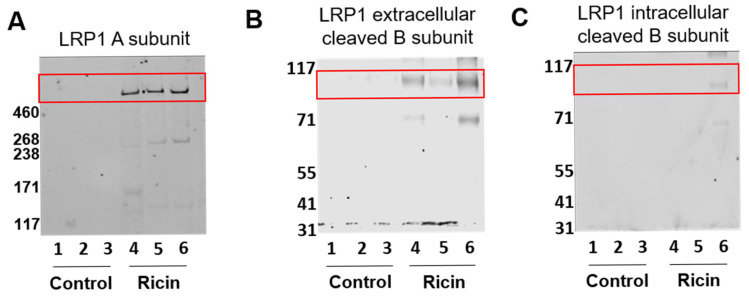
Detection of soluble cleaved LRP1 in BALF of ricin-intoxicated mice. Mice were intranasally exposed to ricin and BALF was collected 72 h post-exposure. Control mice served as controls. The presence of cleaved soluble LRP1 (red box) was assessed using Western Blot analysis with antibodies targeting different domains of LRP1: (**A**) Anti-LRP1 A subunit antibody (**B**) Anti-LRP1 B subunit extracellular domain antibody. (**C**) Anti-LRP1 B subunit intracellular domain antibody (*n* = 3 per group).

To confirm that the LRP1 detected in the BALF of ricin-intoxicated mice and swine represented the soluble, cleaved configuration of LRP1, we performed a comprehensive Western Blot analysis. To define the structural composition of LRP1 in the BALF, we employed three specific antibodies targeting the three distinct segments of the LRP1 protein, the anti-LRP1 A subunit (α-chain) antibody, anti-LRP1 B subunit (β-chain) extracellular domain antibody and anti-LRP1 B subunit (β-chain) membrane-anchored domain antibody. BALF samples were collected 72 h post pulmonary exposure to ricin and analyzed alongside controls. Analysis of BALF samples using the anti-LRP1 A subunit antibody revealed a specific appearance of A subunit in the samples isolated from ricin intoxicated mice (Figure 2A). The antibody in this blot targets the large extracellular α-chain of LRP1, which means that this antibody recognized the shed, soluble form of LRP1 in the BALF. Similarly, Western Blot analysis using the anti-LRP1 B subunit extracellular domain antibody demonstrated an appearance of appropriate band in the BALF of ricin-exposed mice (Figure 2B), because it recognizes the extracellular portion of the β-chain, which is also a part of the shed LRP1. The detected bands by these two antibodies provided a strong evidence for a significant elevation in the levels of cleaved LRP1 protein in the lungs of ricin-exposed mice. This observation was consistent with our earlier findings of increased LRP1 levels in BALF following ricin exposure (Figure 1A,B).

To further characterize the nature of LRP1 present in the BALF, we conducted a Western Blot analysis using an antibody specific to the membrane-anchored domain of the LRP1 B subunit. Notably, no bands were detected in two out of three BALF samples collected from ricin-exposed mice using this antibody (Figure 2C). A weak band was observed in the BALF of the third intoxicated mouse, likely originating from the fragments of dead cell membranes. The absence or minimal expression of bands corresponding to the membrane-anchored domain of the B subunit provided crucial evidence supporting the presence of the cleaved, soluble configuration of LRP1 in the BALF.

### 2.2. The Cleaved Configuration of LRP1 Retained the Ability to Bind Ricin

**Figure 3:**
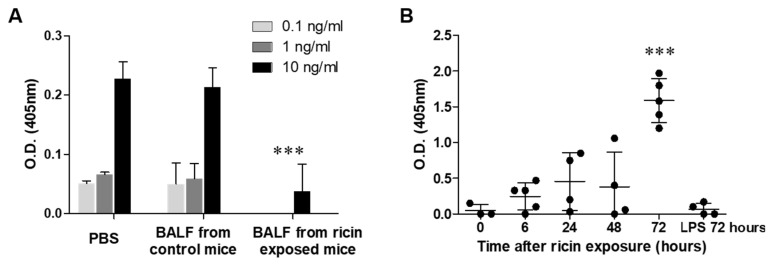
Interaction between ricin and soluble LRP1 in BALF. (**A**) Ricin concentrations were measured by ELISA after incubation of the toxin at concentration 0.1, 1 and 10 ng/mL with either phosphate-buffered saline (PBS), BALF from control mice or BALF from ricin-intoxicated mice isolated 72 h post-exposure. Statistical significance was determined using two-way ANOVA followed by Bonferroni’s post-hoc tests. (**B**) Analysis of ricin-sLRP1 complexes formation by ELISA at different time points following ricin exposure and 72 h post-exposure to LPS. Data are presented as means ± SD (*n* = 4–5 mice per group, each point represents an individual mouse). Statistical significance was determined using one-way ANOVA followed by Tukey’s multiple comparison post-hoc test. *** *p* < 0.001 compared to control mice.

Having established the presence of substantial LRP1 shedding in the lungs of ricin-exposed mice, we sought to determine whether this cleaved LRP1 retained its ability to bind ricin. For this purpose, we developed an assay to assess ricin-binding capacity of components in the BALF of ricin-intoxicated mice. We incubated increasing doses of ricin (0.1, 1, and 10 ng/mL) in PBS (control) or BALF samples isolated from control and ricin exposed mice (72 h post-pulmonary exposure). The samples were then analyzed using an ELISA-based ricin detection assay to determine if any components in the BALF competed with the anti-ricin capture antibody for ricin binding. In both PBS and BALF of control mice, the optical density (O.D.) values increased proportionally with ricin concentration, indicating that neither PBS nor control BALF contained substances that significantly interfered with ricin binding to the capture antibody. In stark contrast, when ricin was incubated with BALF collected from mice 72 h post-exposure to ricin, we observed a significant reduction in ricin detection. At 0.1 and 1 ng/mL ricin, we found no detectable signal and at 10 ng/mL ricin we received a significantly reduced signal compared to control BALF (Figure 3A). These results implied that a soluble factor in the BALF of ricin-intoxicated mice effectively bound ricin, thereby preventing its binding to the capture anti-ricin antibody in the assay. We hypothesized that the soluble protein binding ricin in the BALF of intoxicated mice was sLRP1. To test this possibility, we examined the presence of ricin-sLRP1 complexes. For this purpose, we developed a novel, specific ELISA assay to capture and quantify these complexes. In the developed sandwich ELISA, mouse anti-ricin antibody served as the capture antibody, while rabbit anti-LRP1 antibody was used as the detection antibody. Using this assay, we analyzed BALF samples collected at various time points post-ricin exposure. Ricin-sLRP1 complexes were already detectable in BALF collected at 24 h post exposure, but their levels were not statistically significant compared to BALF of control mice. Significantly elevated levels of Ricin-sLRP1 complexes were found at BALF samples collected 72 h post exposure (Figure 3B). To ensure the specificity of our assay, we tested BALF samples from mice exposed to LPS and no detectable ricin-sLRP1 complexes were observed in these BALF samples (Figure 3B). This result not only confirmed the specificity of the assay for ricin-sLRP1 complexes, but also ruled out the possibility of non-specific interactions or cross-reactivity with other inflammatory mediators with ricin.

### 2.3. Differential Shedding of LPR1 in Lung Cell Populations Following Pulmonary Exposure to Ricin

**Figure 4:**
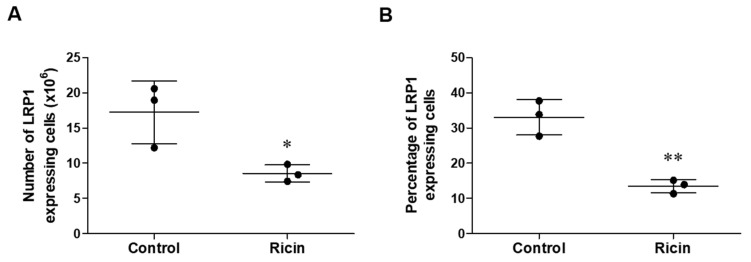
LRP1 expression on total lung cells following ricin exposure. Single-cell suspensions were prepared from lungs of control mice and mice 72 h post-ricin exposure. LRP1 expression was assessed by flow cytometry using an anti-LRP1 A subunit antibody. Data are presented as means ± SD (*n* = 3 mice per group, each point represents an individual mouse). (**A**) Absolute number and (**B**) percentage of LRP1-expressing lung cells. Statistical significance was determined using unpaired *t*-test. * *p* < 0.05, ** *p* < 0.01 compared to control mice.

We previously demonstrated that various lung cell populations exhibit differential rates and intensities of ricin binding, which correlate with their susceptibility to ricin-induced injury [5]. We have identified alveolar macrophages and AT-II cells as the most susceptible lung cell populations to ricin intoxication. This susceptibility is characterized by substantial ricin-induced depurination and cell death following ricin exposure [5,21]. We also found strong correlation between the high levels of LRP1 expression on cells and their ricin susceptibility, as the most susceptible cells to ricin, alveolar macrophages and AT-II cells, predominantly express LRP1 [22]. In this respect, if LRP1 shedding would occur in ricin-susceptible cell populations, it may affect toxin binding and provide a protective effect for these cells. To assess the intensity of LRP1 shedding in lung cells after pulmonary exposure to ricin, we first examined the expression of LRP1 in total lung cells from control mice and mice 72 h post-ricin intoxication by flow cytometry. We quantified the number of lung cells expressing LRP1 (using anti-LRP1 A subunit antibody) in both control and ricin-exposed mice. Statistical analysis revealed a significant decrease in the number and in the percentage of LRP1-expressing cells in ricin-exposed lungs compared to control (Figure 4A,B). The observed decrease in LRP1-expressing cells in the lungs following ricin exposure could be attributed to selective cell death of cells expressing LRP1 and thus preferentially damaged and eliminated during ricin intoxication or due to ectodomain shedding of the LRP1 receptor from cell surfaces, resulting in cells that no longer express detectable levels of membrane-bound LRP1.

**Figure 5:**
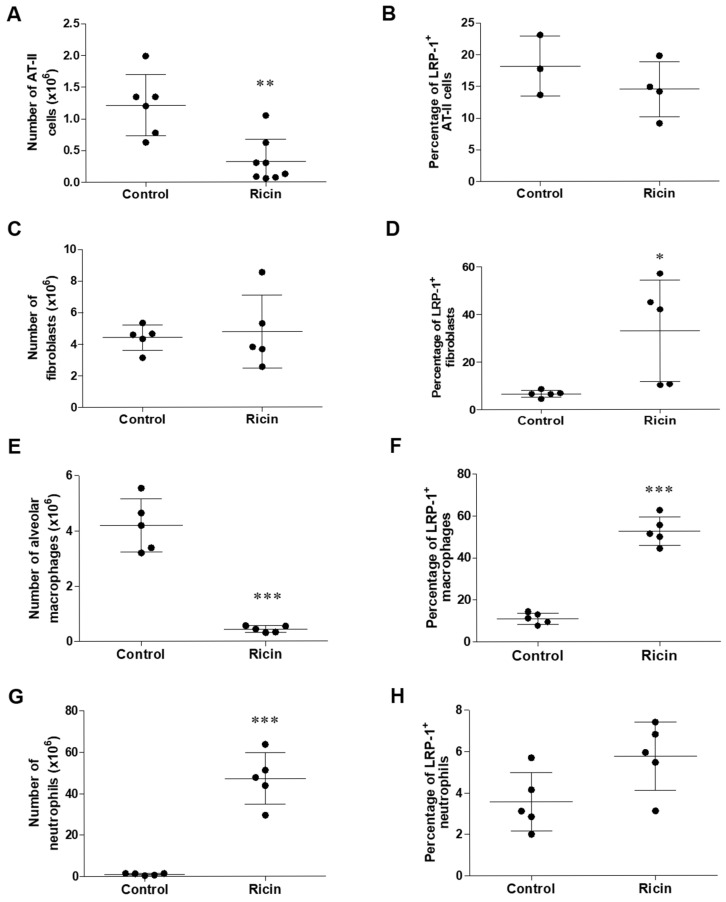
Identification of lung cell populations exhibiting cleaved LRP1 receptor following ricin intoxication. Single-cell suspensions were prepared from the lungs of control mice and mice 72 h post-ricin exposure and the expression of cleaved LRP1 (using anti-LRP1 B subunit antibody) was analyzed by flow cytometry. Graphs presenting total number of (**A**) AT-II, (**C**) fibroblasts, (**E**) alveolar macrophages and (**G**) neutrophils. Graphs presenting percentage of cleaved LRP1 receptor of (**B**) AT-II, (**D**) fibroblasts, (**F**) alveolar macrophages and (**H**) neutrophils. Data are presented as means ± SD (*n* = 3–8 mice per group, each point represents an individual mouse). Statistical significance was determined using unpaired *t*-test. * *p* < 0.05, ** *p* < 0.01, *** p < 0.001 compared to control mice.

**Figure A1:**
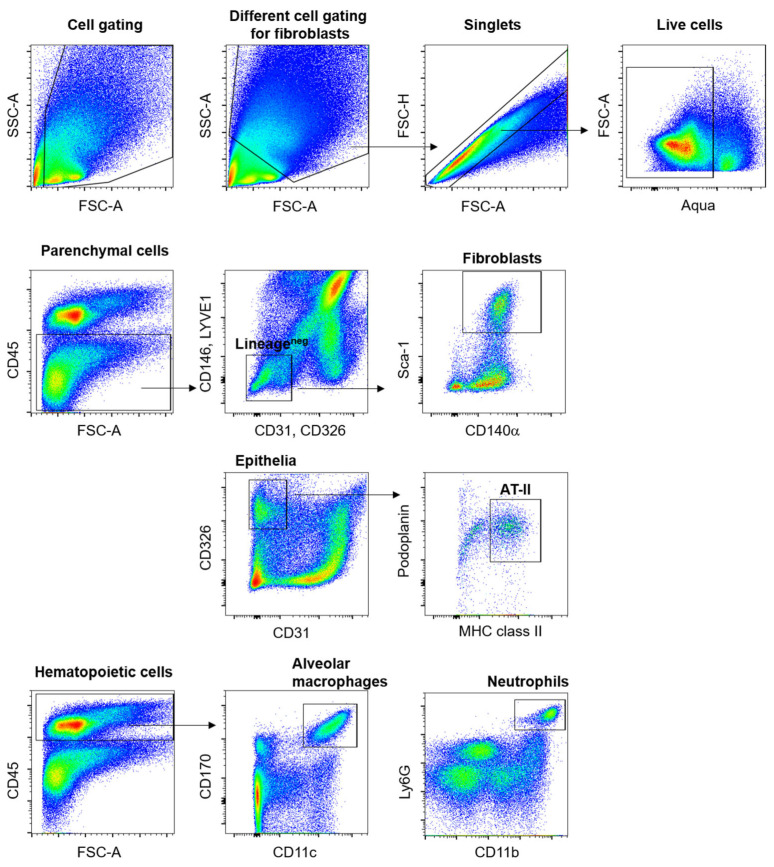
Gating strategy to identify cell populations in the lungs. Cells were gated for singlets and live (Aqua^neg^) cells. Lung parenchymal cells were first defined as CD45^neg^ cells. Then, fibroblasts were gates as CD146, LYVE1, CD31 and CD326^neg^ and Sca-1 and CD140α^pos^. Epithelial cells were gated as CD326^high^ and CD31^neg^ and then, AT-II subpopulation was gated as Podoplanin^high^ and MHC class II^high^ cells. For hematopoietic cell identification, cells were gated for CD45^high^ cells. Alveolar macrophages were gated as CD11c^high^ and CD170^high^ and neutrophils as Ly6G^high^ and CD11b^high^.

To understand the cell-specific dynamics of LRP1 shedding in response to ricin intoxication, we examined various lung cells for the exposed domain that is exposed on the cell membrane after ectodomain shedding by using anti-LRP1 B subunit (carboxy terminus). We determined both the changes in cell numbers and the level of LRP1 shedding in the lungs of control and ricin-intoxicated mice at 72 h post-exposure. This approach allowed us to distinguish between actual receptor shedding and potential artifacts from cell loss or death. We examined parenchymal cell populations, AT-II cells and fibroblasts, and hematopoietic cell populations, such as alveolar macrophages and neutrophils (Figure A1). Notably, among the parenchymal cells, AT-II cells exhibited significant injury from ricin intoxication, as evidenced by a marked decrease in cell number 72 h post-exposure (Figure 5A) in comparison to fibroblasts, which were insensitive to ricin (Figure 5C). Despite the substantial decrease in AT-II cell numbers, we did not observe significant LRP1 shedding in the remaining cells of this population (Figure 5B). Fibroblasts demonstrated resistance to ricin-induced cell death (Figure 5C), but exhibited a significant increase in LRP1 shedding, as evidenced by the elevated percentage of cells expressing the cleaved form of LRP1 on their cell membrane (Figure 5D). Among hematopoietic cell populations, macrophages exhibited severe depletion following ricin exposure, with cell numbers decreasing by an order of magnitude (Figure 5E). Notably, despite this substantial cell loss, we observed a significant increase in the proportion of remaining macrophages undergoing LRP1 shedding. Almost 60% of the surviving macrophages showed evidence of LRP1 ectodomain shedding (Figure 5F). In stark contrast to macrophages, neutrophil showed a dramatic increase in their cell numbers, with a 50-fold rise (Figure 5G) due to massive infiltration following pulmonary ricin intoxication [5]. However, no change in the proportion of neutrophils undergoing LRP1 shedding was observed (Figure 5H). The percentage of neutrophils exhibiting LRP1 shedding remained consistently low in both control and ricin-exposed lungs, suggesting that LRP1 shedding was not a significant feature of the neutrophil response to ricin-induced lung injury. These findings emphasized the complex and cell-specific nature of the response of lung cells to ricin intoxication. The differential patterns of LRP1 shedding across cell types suggested that this process might play varying roles in different cell populations, potentially contributing to their relative susceptibility or resistance to ricin-induced injury.

**Figure 6:**
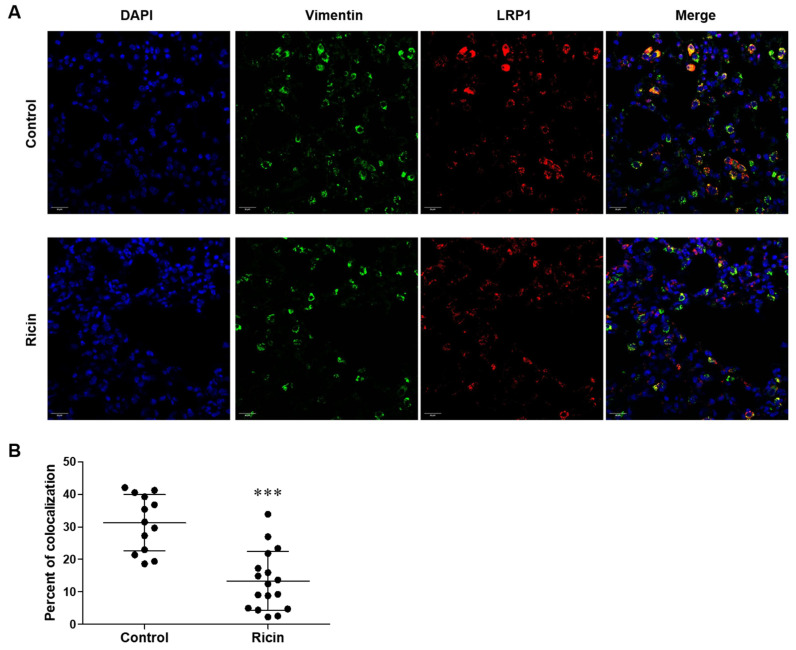
LRP1 expression on lung fibroblasts following ricin intoxication. Lung sections from (**A**) control mice and mice 72 h post-ricin exposure were stained for fibroblasts using anti-vimentin antibody and for intact LRP1 using anti-LRP1 A subunit antibody. Representative images captured by confocal microscope are shown. (Scale bar 10 μm). (**B**) Percentage of colocalization between vimentin and LRP1 staining relative to total vimentin staining. Results represent 5–6 measurements for each lung (*n* = 5, each point represent a measurement of colocalization in the lung), presented as mean ± SD. Statistical significance was determined using unpaired *t*-test. *** *p* < 0.001 compared to control mice.

Observing significant LRP1 ectodomain shedding in lung fibroblasts, along with their relative resistance to ricin-induced cell death, we conducted histological analysis to further characterize changes in LRP1 expression in these cells. We performed immunohistochemical staining of lung sections from control and ricin-exposed mice at 72 h post-exposure employing a dual-staining approach to simultaneously visualize fibroblasts by anti-vimentin antibody and LRP1 expression. We found co-localization of vimentin and LRP1 in the lungs of control mice, which was represented by yellow color in merged images, indicating that, fibroblasts expressed LRP1 on their cell surface (Figure 6A). Consistent with the flow cytometric results, we identified a significant decrease in LRP1-expressing fibroblasts in lungs of intoxicated mice compared to control mice that was evident by the reduction of the yellow color in the merged images (Figure 6A). This decrease was quantified by the percentage of fibroblasts expressing LRP1 based on the co-localization of vimentin and LRP1 staining (Figure 6B). The maintenance of the overall fibroblast population, as indicated by consistent vimentin staining coupled with reduced LRP1 expression might suggest that LRP1 shedding in these cells represented a specific cellular response that protected fibroblasts from ricin binding and death.

### 2.4. Evaluation of Metalloproteinase-Mediated LRP1 Shedding in Fibroblasts

**Figure 7:**
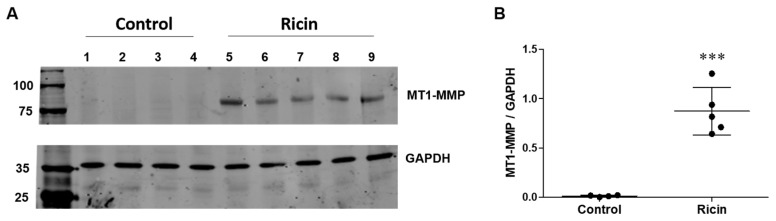
Expression of the metalloproteinase MT1-MMP in mice lungs following ricin intoxication. (**A**) Homogenates of lungs from control mice (samples 1–4) and mice 72 h post-ricin exposure (samples 5–9) were tested for the expression of MT1-MMP by Western Blot analysis. GAPDH was used as a control for protein loading. (**B**) Densitometry analysis of Western Blot results presented as the ratio of MT1-MMP to GAPDH expression for each lung (*n* = 4–5 mice per group, each point represents an individual mouse), mean ± SD. Statistical significance was determined using unpaired *t*-test. *** *p* < 0.001 compared to control mice.

Matrix metalloproteinases (MMPs) have been implicated in the shedding of LRP1 across various cell types [34,36]. Previous research on LRP1 shedding in the lungs of ARDS patients identified membrane type-1 MMP (MT1-MMP) as the primary protease responsible for LRP1 shedding from lung fibroblasts [37]. Since our earlier findings indicated that pulmonary exposure to ricin leads to ARDS development [6], we examined whether MT1-MMP levels increase following ricin exposure. Our analysis revealed a significant elevation in MT1-MMP levels in lung homogenates at 72 h post-ricin exposure compared to control mice (Figure 7A,B). The upregulated levels MT1-MMP in lungs of ricin intoxicated mice might contribute to the increase of soluble LRP1.

**Figure 8:**
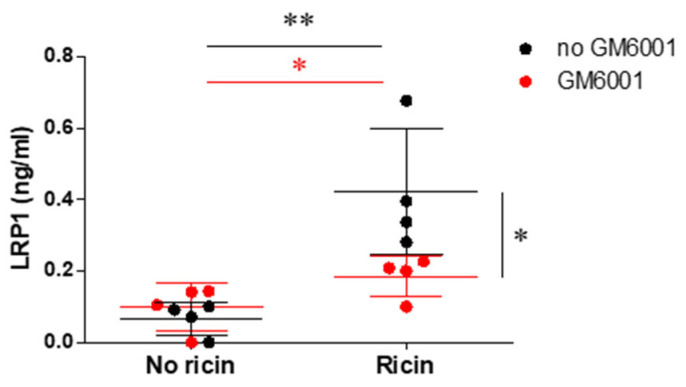
sLRP1 levels in fibroblast cell line following ricin intoxication in absence or presence of metalloproteinase inhibitor. MEFs were incubated for 1 h with ricin (100 ng/mL) in presence (red dots) or absence (black dots) of the metalloproteinase inhibitor GM6001 (100 µM). Twenty-four hours after incubation, sLRP1 levels in the cell medium were measured using a commercial ELISA kit. Data are presented as means ± SD (*n* = 3–5 experiments per group, each point represents an individual experiment). Statistical significance was determined using two-way ANOVA followed by Bonferroni’s post-hoc tests. * *p* < 0.05, ** *p* < 0.01 compared to control cells (no ricin).

To further understand the mechanism of LRP1 shedding, we examined the secretion of soluble LRP1 in a controlled in vitro system using Mouse Embryonic Fibroblasts (MEF). This approach allowed us to directly measure soluble LRP1 release and to define the role of metalloproteinases in the shedding process of LRP-1. For this purpose, MEF cells were exposed to ricin in the absence or presence of GM6001 (Ilomastat), a potent broad-spectrum metalloproteinase inhibitor. We measured the concentration of soluble LRP1 in the cell culture medium using ELISA assay. As shown in Figure 8, we observed a significant elevation of soluble LRP1 in the medium of ricin-exposed MEF cells that were not treated with GM6001, which confirmed that ricin intoxication directly induced LRP1 shedding in fibroblasts. Then, treatment with GM6001 substantially reduced the release of sLRP1 from intoxicated cells. This result implied that ricin exposure activated or upregulated the expression of metalloproteinases responsible for LRP1 ectodomain cleavage.

## 3. Discussion

The existence of various soluble components derived from membrane receptors has been well-documented, and in certain instances, the mechanisms underlying their shedding have been elucidated [42,43]. These soluble receptor components can modulate receptor activity in diverse ways. For example, soluble TNF-alpha receptors act as antagonists, inhibiting the activity of their membrane-bound counterparts. Conversely, soluble IL-6 receptors function as agonists, enhancing the activity of the membrane receptor [43]. More complex scenarios are exemplified by the soluble IL-4 receptor, which can either antagonize or agonize the activity of the membrane receptor depending on the availability of its ligand [44]. However, not all soluble receptors exert significant influence over their membrane-bound forms. Some soluble receptors have binding constants that are relatively low compared to their membrane-bound counterparts, resulting in negligible impact on the overall receptor activity [43]. This dual functionality underscores the intricate regulatory roles that soluble receptors can play in cellular signaling pathways.

Numerous studies have demonstrated that LRP1 receptors undergo shedding from the membrane through the action of various metalloproteinases [34,35,36]. This process results in the release of a soluble form of LRP1 (sLRP1), which includes the entire α subunit and a portion of the β subunit of the receptor [39,40]. The presence of sLRP1 has been detected in several biological fluids, including plasma [45], cerebrospinal fluid [46], and lung fluid in various inflammatory conditions, such as ARDS [37], rheumatoid arthritis, and systemic lupus erythematosus [47]. These findings suggest that sLRP1 may serve as a biomarker for inflammation and potentially play a role in disease progression. The shedding of LRP1 and its impact on the activity of the membrane-bound receptor remains a subject of ongoing investigation. This question becomes particularly complex in the context of pulmonary ricin intoxication since LRP1 not only contributes significantly to lung inflammation, but also functions as the primary receptor for ricin in the lungs [22]. The dual role of LRP1 in this scenario presents a unique challenge in understanding the implications of its shedding.

In this study, we observed that levels of soluble LRP1 (sLRP1) significantly increased in the lungs of both mice and swine following pulmonary exposure to ricin. The comparison with LPS-induced acute lung injury revealed that ricin intoxication provoked a markedly stronger LRP1 shedding response. The approximately two-fold increase in sLRP1 levels observed in ricin-exposed lungs compared to LPS-treated counterparts suggests that ricin activates more robust receptor cleavage mechanisms. This heightened response may be attributed to the more pronounced inflammatory burden elicited by ricin, along with increased secretion or activation of metalloproteinases in the lungs, key enzymes involved in ectodomain shedding. Notably, the low levels of sLRP1 detected in the plasma of intoxicated mice suggest that the source of sLRP1 is the lung tissue itself. This observation is consistent with findings in patients with ARDS, where elevated levels of sLRP1were detected in the lungs, but not in the plasma [37]. Furthermore, we observed a correlation between the presence of ricin in the lungs and the shedding of LRP1. We found that mice treated with anti-ricin antibodies exhibited a marked decrease in sLRP1 levels compared to untreated mice. This reduction in sLRP1 levels was consistent regardless of the timing of antibody administration post-exposure. This suggests that antibody treatment extends beyond toxin neutralization and may also play a role in modulating the inflammatory response and cellular processes associated with LRP1 shedding.

Many studies have demonstrated that the soluble form of the LRP1 receptor retains its ability to bind the natural ligands of the membrane-bound receptor, but lacks the capacity to internalize these ligands via transcytosis. Consequently, sLRP1 competes with the membrane-bound LRP1 for ligand binding, effectively acting as a decoy receptor [39,40]. For instance, it has been shown that sLRP1 competes with membrane-bound LRP1 for binding to TIMP-3, and the sLRP1-TIMP-3 complexes retain their ability to inhibit metalloproteinases [48]. Another example is the binding of sLRP1 to MMP-13, which protects MMP-13 from degradation within cells, thereby allowing it to remain active in the extracellular space [49]. In this study, we have demonstrated the ability of soluble LRP1 to bind ricin by identifying sLRP1-ricin complexes in the BALF of intoxicated mice. This novel finding has significant implications for our understanding of ricin toxicity and the potential for therapeutic interventions. The formation of sLRP1-ricin complexes suggests that the binding of ricin to the soluble receptor may protect the toxin from degradation and clearance from the lungs. This protection could potentially prolong the availability of active ricin in the pulmonary environment, enabling it to intoxicate additional cells over an extended period. This hypothesis is supported by our previous observations that mice can be rescued from ricin intoxication by anti-ricin antibody treatment administered as late as 48 h post-exposure. Furthermore, our earlier work demonstrated that the depurination process caused by ricin continues to occur in the lungs even at late time points post-exposure (48–72 h) [21]. This finding provides additional evidence for the presence of active ricin in the lungs long after the initial exposure, aligning with the concept of sLRP1-mediated toxin preservation.

The shedding of LRP1 is known to be a selective process, occurring differentially across various cell types [37,38]. In the context of pulmonary ricin intoxication, identifying the specific cell populations undergoing LRP1 shedding is critical for understanding its role in disease progression. Our previous research has highlighted alveolar macrophages and epithelial type II cells, as the primary lung cell populations affected by ricin intoxication. Predominant shedding of LRP1 in these susceptible cell populations could potentially serve a protective function by reducing ricin internalization. Therefore, we investigated the cell-specific dynamics of LRP1 shedding following pulmonary ricin exposure and revealed intriguing patterns across different lung cell populations. First and foremost, we observed a significant increase in LRP1 shedding specifically in lung fibroblasts, a cell population that, for the first time in this study, was shown to be resistant to ricin-induced cytotoxicity. This finding was corroborated by immunohistochemical analysis of lung sections from intoxicated mice, which showed a marked decrease in LRP1 expression on fibroblasts. Interestingly, among the parenchymal cell populations examined, fibroblasts were unique in exhibiting this elevated LRP1 shedding response. AT-II cells did not show significant changes in LRP1 shedding. The predominant shedding of LRP1 in fibroblasts aligns with observations in ARDS patients, where a similar pattern was noted without involvement of epithelial cells in the shedding process [37]. This parallel suggests that LRP1 shedding in fibroblasts may be a common response in severe lung injury, regardless of the initial insult. In fibroblasts, LRP1 acts as a key regulator of ECM remodeling by inhibiting the remodeling of fibronectin. This inhibitory effect is primarily mediated through the regulation of cell-surface urokinase receptor (uPAR) and plasminogen activation by urokinase-type plasminogen activator (uPA). LRP1 deficiency in fibroblasts leads to increased fibronectin remodeling, highlighting its importance in maintaining ECM homeostasis [50]. The role of LRP1 in fibroblasts extends beyond ECM remodeling. It is involved in various signaling pathways that regulate fibroblast differentiation and transdifferentiation [51]. In fibrotic tissues, such as those found in the skin and lungs of Fra-2 transgenic mice, LRP1 depletion is associated with c-Jun overexpression and fibrotic changes [52]. These findings raise important questions about the role of LRP1 shedding in fibroblasts during ricin-induced lung injury. It is possible that this process serves a protective function, potentially by modulating the inflammatory response or altering the susceptibility of fibroblasts to ricin toxicity. Alternatively, it could contribute to the pathogenesis of lung injury by altering the extracellular matrix or fibroblast function.

Our investigation into the hematopoietic cell populations following pulmonary exposure to ricin revealed significant LRP1 shedding in alveolar macrophages. LRP1 is abundantly expressed in macrophages and is mainly associated with suppression of inflammation. Suppressive effects are demonstrated by the ability of the LRP1-expressing cells to engulf apoptotic cells (efferocytosis) and inhibit the production of inflammatory proteins [53,54,55]. The ectodomain shedding of LRP1 in macrophages has been implicated in various inflammatory conditions [34,38]. However, given the substantial depletion of alveolar macrophages from the lung post-intoxication, the relevance of this shedding process to the overall pathology of ricin-induced lung injury appears limited.

In the ectodomain shedding process, two primary families of proteases are involved, matrix metalloproteinases (MMPs) and a disintegrin and metalloproteinase (ADAM) family [56]. In this study, we employed GM6001, a broad-spectrum metalloproteinase inhibitor, to investigate the inhibition of ectodomain shedding. Our results demonstrated that GM6001 effectively inhibited the shedding of LRP1 in a fibroblast cell line following exposure to ricin. This finding suggests that the proteolytic activity mediated by metalloproteinases is responsible for the increased shedding of LRP1 observed after ricin exposure. Additionally, our findings revealed an increase in MT1-MMP levels in the lungs of ricin-intoxicated mice, which we hypothesize is responsible for the fragmentation of the LRP1 receptor. This observation aligns with previous studies that have demonstrated the involvement of MT1-MMP in LRP1 shedding in ARDS patients [37]. However, it is important to note that our results do not preclude the involvement of other metalloproteinases in the ectodomain shedding process within the lungs. For instance, ADAM17 has been implicated in LRP1 shedding in various tissues [56], and its potential role in ricin-induced LRP1 shedding in the lungs cannot be discounted.

It is not fully understood why the shedding of LRP1 in the lungs following pulmonary exposures occurs specifically in the fibroblast population. However, it appears that inhibiting this shedding has a beneficial effect on cell survival. LRP1 is known to act as an anti-inflammatory inhibitor in several pathologies by regulating the expression of proteases and growth factors involved in inflammatory processes [38]. LRP1-deficient cells exhibit high activation levels of NF-κB and increased secretion of TNF-α [57]. In a mouse model of atherosclerosis, LRP1 was shown to inhibit the expression of inflammatory mediators such as CCL2 and MMP-9 [58].

Conversely, the presence of soluble LRP1 (sLRP1) enhances the inflammatory response, primarily due to the delayed clearance of MMP-2 and MMP-9 from the membrane receptor. Elevated levels of these two metalloproteinases have been observed in the BALF of ARDS patients and in animal models of acute lung injury, with their levels positively correlating with sLRP1 levels [59,60,61]. Our previous work demonstrated a significant increase in MMP levels in the lungs of mice exposed to ricin via pulmonary routes [62]. Research has highlighted the detrimental effects of high MMP levels in the lungs. In mouse models of acute lung injury, MMP-deficient mice exhibited less lung damage compared to wild-type mice [63,64]. MMPs, particularly MMP-2 and MMP-9, cause rapid reduction of the junction proteins leading to disruption of alveolar-capillary barrier, a phenomenon observed in the lungs of mice shortly after ricin exposure [62]. This process hinders the healing process and promotes tissue repair through fibrotic pressure [65].

Additionally, the release of soluble LRP1 (sLRP1) into the extracellular space appears to have far-reaching consequences beyond the immediate effects on LRP1-mediated signaling by inhibiting the endocytosis of other crucial ligands of the membrane-bound LRP1 receptor such as urokinase (uPA), tissue-type plasminogen activators (tPA), thrombospondin and fibronectin. These molecules play pivotal roles in various aspects of the inflammatory response, including extracellular matrix remodeling, cell adhesion, and proteolytic cascades [66,67,68]. By interfering with their endocytosis, LRP1 shedding may lead to their accumulation in the extracellular space, potentially exacerbating inflammatory conditions.

This work provides new perspectives on the role of LRP1 in pulmonary ricin intoxication, opening avenues for novel anti-inflammatory strategies. Specifically, interventions aimed at preventing LRP1 shedding could potentially mitigate the inflammatory response associated with ricin exposure. However, given the complex and potentially dual role of LRP1, further research is needed to fully elucidate the consequences of inhibiting LRP1 shedding and to develop targeted therapeutic approaches.

## 4. Materials and Methods

### 4.1. Animal Ethics

All animal experiments were conducted in compliance with the Israeli law and approved by the Institutional Animal Care and Use Committee (IACUC) at the Israel Institute for Biological Research under protocol numbers P-04-16, M-06-19 and M-07-20. Animals were monitored daily during intoxication experiments. Mice exhibiting two or more of the following signs were considered for euthanasia: matted hair, hunched posture, breathing difficulty, and tremors. Swine displaying peripheral cyanosis or not approaching food and water for 24 h were also considered for euthanasia.

### 4.2. Animals

Mice: female CD-1 mice (27–32 g) were purchased from Charles River Laboratories Ltd. (Margate, UK). Mice were housed in filter-top cages in an environmentally controlled room and maintained at 21 ± 2 °C and 55 ± 10% humidity. Lighting was set to mimic a 12/12 h dawn to dusk cycle. Mice were housed in a purpose-built animal holding facility for 4–8 days prior to the beginning of the experiment. Animals were allowed access to water and food ad libitum. Swine: female pigs (Topigs 20, 15–24 kg, aged 7–9 weeks), were obtained from an approved commercial source (Van Beek, Drunen, The Netherlands). Pigs were fed a standard commercial pig diet at 4% body weight per day and housed in a purpose-built animal holding facility for 4–8 days prior to the beginning of the experiment. Animals were allowed access to water ad libitum through automatic water nipples.

### 4.3. Ricin Preparation

The use of ricin in the current study and purification of ricin from castor beans were conducted under the safety and environmental regulations of the Israel Institute for Biological Research, in compliance with the Israeli law. Crude ricin was prepared from seeds of endemic Ricinus communis, as previously described [3]. Shortly, seeds were homogenized in a blender (Waring, Torrington, CT, USA) in 5% acetic acid (Merck, Darmastadt, Germany)/PBS (Biological Industries, Beth-Haemek, Israel). The homogenate was centrifuged, and the clarified supernatant containing the toxin was subjected to ammonium sulfate (Merck, Darmastadt, Germany) precipitation (60% saturation). The precipitate was dissolved in PBS and dialyzed extensively against the same buffer. The toxin preparation appeared on a Coomassie blue (Bio-Rad, Rishon Le Zion, Israel)-stained nonreducing 10% polyacrylamide gel (ThermoFisher Scientific, Carlsbad, CA, USA) as two major bands of molecular mass of ~65 kDa (ricin toxin, ~80%) and 120 kDa (Ricinus communis agglutinin, ~20%). Protein concentration was determined as 2.86 mg/mL by 280-nm absorption (Nanodrop 2000; ThermoFisher Scientific, Waltham, MA, USA).

### 4.4. Animal Intoxication and Treatment

Mice: prior to intoxication, mice were anesthetized by an intraperitoneal injection of ketamine (1.9 mg/mouse, Vetoquinol, Lure, France) and xylazine (0.19 mg/mouse, Eurovet Animal Health, AD Bladel, The Netherlands). Subsequently, crude ricin (50 µL, 2LD_50_, 10 µg/kg) or LPS (50 µL, 4 mg/kg) was applied intranasally.

Treatment of ricin-intoxicated mice was carried out using an anti-ricin antibody comprising purified polyclonal F(ab)2 antibody-fragments of equine source. Specifically, mice were intravenously treated at the indicated time-points with the anti-ricin F(ab)2 (100 µL, 60 mg/mL). Swine: prior to intoxication, pigs were anesthetized with intramuscular ketamine and xylazine (10 and 1 mg/kg, respectively) and thereafter an ear vein was punctured and intravenous anesthesia was deepened with propofol (1%, 1–2 mL), immediately followed by intubation with a cuffed endotracheal tube (5.5–6.0 mm internal diameter). Intratracheal penetration was verified by capnography and localization of the distal tube-end above the tracheal bifurcation was verified by chest X-ray. Crude ricin (2LD_50_, 3 µg/mL/kg) divided into two portions was instilled to the raised (~30°) supine pig while tilting the animal from right to left.

### 4.5. BALF Collection

Mice: BALF was collected by instillation of 1 mL PBS at room temperature via a tracheal cannula immediately thereafter recovering the fluid with the same syringe. Swine: BALF was collected by intratracheal insertion of a Metrix feeding tube (ConvaTec, Sunderland, UK) through a cuffed endotracheal tube (Well Lead, Guanguhou, China) and instillation of 30 mL PBS immediately thereafter recovering the fluid with a 50 mL syringe. BALF was centrifuged at 240× *g* at 4 °C for 10 min and supernatants were stored at −20 °C until analysis.

### 4.6. Western Blot Analysis

Lungs were perfused with ice-cold PBS and homogenized in RIPA buffer (Sigma-Aldrich, St. Louis, MO, USA, Cat. No. R0278), supplemented with protease inhibition cocktail (Sigma-Aldrich, St. Louis, MO, USA, Cat. No. P8340) and Halt protease inhibitor cocktail (Thermo Scientific, Waltham, MA, USA, Cat. No. 87786) using a PolyTron homogenizer (Kinematica, Model P2100, Luzern, Switzerland). Homogenates were incubated for 2 h at 40 °C and clarified by centrifugation at 15,000× *g* for 20 min. Protein concentration was determined using the Bradford reagent (Bio-Rad, Hercules, CA, USA, Cat. No. 5000006). BALF or lung lysates were diluted to 20 µg/mL with Laemmli sample buffer supplemented with DTT (Bio-Rad, Hercules, CA, USA, Cat. No. 1610747) and heated to 95 °C for 5 min. Lysates were resolved by 3–8% Tris-Acetate SDS gels (Invitrogen, Waltham, MA, USA, Cat. No. EA0378BOX) and transferred onto nitrocellulose membranes (Invitrogen, Waltham, MA, USA, Cat. No. IB301002). Membranes were blocked with 5% nonfat Blotting grade blocker (Bio-Rad, Hercules, CA, USA, Cat. No. 1706404) in Tris-buffered saline/Tween 20 for 1 h at room temperature. Membranes were incubated overnight at 4 °C with the following primary antibodies: rabbit anti-LRP1 N terminal (Sigma-Aldrich, St. Louis, MO, USA, Cat. No. L2295, 1:250), mouse anti-LRP1 cleaved fragment (extracellular domain, Molecular Innovations, Novi, MI, USA, Cat. No. MA5A6719, 1:500), mouse anti-LRP1 (intracellular domain, R&D Systems, Minneapolis, MN, USA, Cat. No. MAB6360, 1:500), anti-MT1-MMP (OriGene, Rockville, MD, USA, Cat. No. TA312180, 1:100), or anti-GAPDH (Cell Signaling Technology, Danvers, MA, USA, Cat. No. 2118S, 1:1000). After washing, membranes were incubated with IRDye 680RD Goat anti-rabbit (LI-COR, Lincoln, NE, USA, Cat. No. 926-68071, 1:20,000) or IRDye 680RD Goat anti-mouse (LI-COR, Lincoln, NE, USA, Cat. No. 926-68070, 1:20,000) for 1 h at 4 °C. Membranes were developed using an ODYSSEY CLx imaging system (LI-COR Biosciences, Lincoln, NE, USA). Band intensities were quantified using Fiji software (version 2.15.0, Rasband, W.S., ImageJ, U.S. National Institutes of Health, Bethesda, MD, USA, 2017).

### 4.7. ELISA Analysis

#### 4.7.1. LRP1 ELISA

Concentrations of LRP1 were determined using a specific enzyme-linked immunosorbent assay (LRP1 ELISA kit, Cat. No. OKEH01469, detection range 0.156–10 ng/mL, Aviva Systems Biology, San Diego, CA, USA) according to the manufacturer’s instructions.

#### 4.7.2. Ricin ELISA

Microtiter plates (Nunc MaxiSorp, Thermo Fisher Scientific, Waltham, MA, USA, Cat. No. 44-2404-21) were coated with rabbit polyclonal anti-ricin antibody (produced in-house, 2.5 μg/mL) diluted in 50 mM NaHCO_3_ buffer and incubated overnight at 4 °C. Plates were blocked using PBST buffer (2% BSA, 0.05% Tween 20, 0.05% Na-Azide in PBS) for 1 h at 37 °C. BALF samples were added to the plate and incubated for 1 h at 37 °C. After washing with PBST, plates were incubated with equine polyclonal anti-ricin F(ab)2 (RR003, 1200 Israeli Neutralizing Units (INU)/mL) for 1 h at 37 °C. Plates were washed again and incubated with anti-equine alkaline phosphatase (AP, 1:1000) conjugate (Sigma-Aldrich, St. Louis, MO, USA, Cat. No. A3169) for 20 min. After a final wash, AP substrate (Sigma-Aldrich, St. Louis, MO, USA, Cat. No. F4523) was added and the reaction was measured at 405 nm using a Spectramax ABS microplate reader (Molecular Devices, Sunnyvale, CA, USA).

#### 4.7.3. ELISA for LRP1-Ricin Complexes

Microtiter plates (Nunc MaxiSorp, Thermo Fisher Scientific, Waltham, MA, USA, Cat. No. 44-2404-21) were coated with mouse anti-ricin antibody mouse (produced in-house, anti-RTB, 2.5 μg/mL) diluted in 50 mM NaHCO_3_ buffer and incubated overnight at 4 °C. Plates were blocked using PBST buffer (2% BSA, 0.05% Tween 20, 0.05% Na-Azide in PBS) for 1 h at 37 °C. BALF samples were added to the plate and incubated for 1 h at 37 °C. After washing with PBST, plates were incubated with rabbit anti-LRP1 N-terminal antibody (Sigma-Aldrich, St. Louis, MO, USA, Cat. No. L2295, 1:200) for 1 h at 37 °C. Then, plates were washed again and incubated with anti-rabbit AP conjugate (Sigma-Aldrich, St. Louis, MO, USA, Cat. No. A3687, 1:1000) for 20 min. After a final wash, AP substrate (Sigma-Aldrich, St. Louis, MO, USA, Cat. No. F4523) was added and the reaction was measured at 405 nm using a Spectramax ABS microplate reader (Molecular Devices, Sunnyvale, CA, USA).

### 4.8. Flow Cytometric Analysis

Lungs were harvested, cut into small pieces and digested for 2 h at 37 °C with 4 mg/mL collagenase D (Roche, Mannheim, Germany) in PBS Ca^+2^ Mg^+2^ (Biological Industries, Beit Haemek, Israel). The tissue was meshed through a 70 µm cell strainer and red blood cells were lysed with red blood cell lysis buffer (Sigma-Aldrich, Rehovot, Israel). Cell suspensions were blocked by incubation with anti-mouse CD16/32 (eBioscience, San Diego, CA, USA, 16-0161-85) on ice for 10 min. Then, cells were stained with the following fluorophore conjugated antibodies: CD45 (clone 30-F11), CD11c (N418), MHC class II (M5/114.15.2), Ly6G (1A8), CD170 (S17007L), CD11b (M1/70), CD31 (390), CD326 (G8.8), Podoplanin (8.1.1), CD146 (ME-9F1), LYVE1 (ALY7), Sca-1 (D7), FITC-coupled Streptavidin and CD140α (APA5). Antibodies were purchased from BioLegend (San Diego, CA, USA), BD Biosciences (Franklin Lakes, NJ, USA) or eBioscience (San Diego, CA, USA). For LRP1 staining and cleaved fragment of LRP1, anti-LRP1 (Sigma, St. Louis, MO, USA, L2295) and anti-LRP1 (Cell Signaling Technology, Danvers, MA, USA, 64099), respectively and Alexa Fluor 647-coupled goat anti-rabbit (Invitrogen, Waltham, MA, USA, A21245) were used. For dead cell exclusion, Aqua Live/Dead cell stain (ThermoFisher, Carlsbad, CA, USA) was used. Cells were collected by flow cytometry using LSR-Fortessa (BD Biosciences, San Jose, CA, USA) and analyzed by FlowJo software (version 10, Tree Star, Ashland, OR, USA).

### 4.9. Immunohistochemistry

Lungs perfused with PBS were harvested and immersed in neutral 4% buffered formalin for 2 weeks at room temperature prior to embedding. Sections (5 µM) were mounted on glass slides and deparaffinized. Antigen retrieval was performed by incubation in Target Retrieval Solution (DAKO, Glostrup, Denmark, 30 min, 95 °C). After blocking in 5% BSA in PBS, slides were incubated (overnight, 4 °C) with anti-LRP1 (Sigma, St. Louis, MO, USA, L2295, 1:100) and anti-Vimentin antibody (Invitrogen, Waltham, MA, USA, PA1-10003, 1:100). Staining with primary antibodies was followed by incubation with Alexa Fluor 594-coupled donkey anti-rabbit and Alexa Fluor 488-coupled goat anti-chicken (Invitrogen, Waltham, MA, USA, A21207, A11039, both diluted 1:100). For nuclear staining, slides were mounted with Prolong^®^ Gold antifade reagent containing DAPI (Invitrogen, Waltham, MA, USA, P36935). Analysis was performed using an LSM 710 confocal microscope (Carl Zeiss, Oberkochen, Germany) and colocalization analysis was calculated using Fiji software (version 2.15.0, Rasband, W.S., ImageJ, U.S. National Institutes of Health, Bethesda, MD, USA, 2017).

### 4.10. Mouse Embryonic Fibroblasts

MEF cell line was purchased from ATCC (SCRC-1008, Manassas, VA, USA). Cells were cultured in Dulbecco’s modified Eagle’s medium (DMEM, Biological Industries, Beit Haemek, Israel) supplemented with 10% fetal bovine serum (FBS), 2 mM L-glutamine, 1 mM sodium pyruvate, and 100 μM non-essential amino acids. Cells were cultured in a humidified incubator at 37 °C with 5% CO_2_. For the cytotoxicity studies, cells were seeded in 96-well plates (5 × 10^4^ cells/well) and incubated with ricin (100 ng/mL) for 1 h in the presence or absence of metalloproteinase inhibitor GM6001 (100 µM, Abcam, Cambridge, UK). 24 h post incubation with ricin, sLRP1 levels in the medium were measured using a commercial ELISA kit (LRP1 ELISA kit, OKEH01469, AVIVA systems, San Diego, CA, USA).

### 4.11. Statistical Analysis

Simple comparisons were performed using the unpaired two-tailed Student’s *t*-test. For multiple comparisons, one-way or two-way ANOVA with Tukey or Bonferroni’s multiple comparison tests were applied. Significance was set at *p* < 0.05. Statistical analysis was calculated using Prism software (version 5.01, 2007; GraphPad Software, La Jolla, CA, USA). All data are presented as means ± SD.

## 5. Conclusions

This study provides new insights into the complex cellular mechanisms underlying ricin-induced lung injury, particularly the role of LRP1 shedding as a potential modulator of toxin susceptibility and inflammatory responses. We show that ricin exposure induces substantial shedding of LRP1 in the lungs, resulting in elevated levels of soluble LRP1 in BALF of both murine and porcine models. Notably, sLRP1 retains ricin-binding capacity, which may serve a dual role—either acting protectively by sequestering the toxin and limiting its uptake by cells, or prolonging toxin presence in the system via a depot effect that delays clearance of the toxin. A key finding is the differential LRP1 shedding among lung cell types. Alveolar type II epithelial cells, highly sensitive to ricin and critical for alveolar homeostasis, exhibited minimal LRP1 shedding, possibly maintaining surface receptor expression and thus enhancing vulnerability to toxin uptake. In contrast, fibroblasts, which were resistant to ricin, showed elevated LRP1 shedding, suggesting a protective mechanism that limits toxin entry. Conversely, although significant LRP1 shedding was observed in alveolar macrophages, their rapid depletion following ricin intoxication suggests that they are unlikely to contribute substantially to the overall LRP1 pool in the lungs. Interestingly, the consistent decrease in BALF LRP1 levels following antibody treatment at various time points indicates that neutralizing ricin diminishes the trigger for LRP1 shedding, even when administered during later stages of intoxication. This supports the hypothesis that ongoing ricin activity drives continued LRP1 shedding and suggests that sLRP1-ricin complexes may shield the toxin from degradation and clearance. Together, these findings underscore the complex, cell-type-specific dynamics of LRP1 shedding in shaping the outcome of ricin-induced lung injury. However, the functional significance of these interactions in modulating toxin distribution and cellular uptake requires further investigation.

## Data Availability

The raw data supporting the conclusions of this article will be made available by the authors on request.

## Abbreviations

The following abbreviations are used in this manuscript:

LRP1	low-density lipoprotein receptor-related protein 1
AT-II	Alveolar epithelial type II
RTA	Ricin toxin A (subunit)
RTB	Ricin toxin B (subunit)
Gb3	Globotriaosylceramide
TEM8	Tumor endothelial marker 8
CMG2	Capillary morphogenesis protein 2
PEA	Pseudomonas aeruginosa exotoxin A
kDa	Kilodalton
ARDS	Acute respiratory distress syndrome
BALF	Bronchoalveolar lavage fluid
sLRP1	Soluble LRP1
LPS	Lipopolysaccharide
MMPs	Matrix metalloproteinases
MT1-MMP	Membrane type-1 MMP
MEF	Mouse Embryonic Fibroblasts
ECM	Extracellular matrix
uPAR	urokinase receptor
uPA	urokinase-type plasminogen activator
ADAM	a disintegrin and metalloproteinase
